# Protective Effects of Resveratrol Supplementation on Contusion Induced Muscle Injury

**DOI:** 10.7150/ijms.35977

**Published:** 2020-01-01

**Authors:** Yi-Ju Hsu, Chun-Shen Ho, Mon-Chien Lee, Chin-Shan Ho, Chi-Chang Huang, Nai-Wen Kan

**Affiliations:** 1 Graduate Institute of Sports Science, National Taiwan Sport University, Taoyuan 33301, Taiwan;; 2 Division of Physical Medicine and Rehabilitation, Lo-Hsu Foundation, Inc., Lotung Poh-Ai Hospital, Yilan 26546, Taiwan.; 3 Center for General Education, Taipei Medical University, Taipei 11031, Taiwan.

**Keywords:** mass-drop injury, regeneration, NSAID, LDH, CK

## Abstract

Muscle injuries frequently occur in contact sports events. The current treatment options for soft tissue injuries remain suboptimal and often result in delayed or incomplete recovery of damaged muscles. Resveratrol (RES) is a phenolic phytochemical, well-known for its antioxidant and anti-inflammatory properties. The purpose of this study is to evaluate the potential beneficial effects of RES supplementation on inflammation and regeneration in skeletal muscle after a contusion injury, in comparison to a conventional treatment of nonsteroidal anti-inflammatory drugs (NSAID). After one week of acclimation, forty eight -week-old male ICR mice were randomly divided into the five groups (n=8 per group): 1) normal control (NC), 2) mass-drop injury without any treatment (mass-drop injury, MDI), 3) post-injury NSAID treatment (MDI+ 10mg/kg NSAID), 4) post-injury RES supplementation (MDI+ 25mg/kg/day RES) and 5) post-injury treatment with RES and NSAID (MDI + resveratrol+ NSAID). After muscle contusion injury of the left gastrocnemius muscle, RES or NSAID were orally administered post-injury once a day for 7 days. Results showed that the MDI group had significantly higher serum uric acid (UA), CREA (creatinine), LDH (lactic dehydrogenase) and creatine kinase (CK) than the normal control group. Treatment with resveratrol reduced muscle damage as evidenced by the significantly decreased serum levels of UA, CREA, LDH and CK after contusion-induced muscle injuries in mice. In addition, RES and RES + NSAID groups promoted muscle satellite cell regeneration with increase in desmin protein after injury. Our results suggest that resveratrol combined with NSAID potentially improve muscle recovery and may be a potential candidate for further development as an effective clinical treatment for muscle repair.

## Introduction

Muscle lesions are the most common forms of injuries in athletes with an incidence rate of 10% to 55% among all sports injuries 1. Sports-related muscle injuries in the lower extremities cause significant opportunity loss in training or competition, affecting exercise performance. The acute soft tissue injury is generally induced by external stress above a threshold, causing localized swelling, pain, bruising, blood capillary dilation, leukocyte infiltration, edema, and hemorrhage 2, 3. In this phase, neutrophils and macrophages rapidly invade injured muscle tissue and provide several important functions contributing to removal of damaged tissue, inflammation and healing 4. Interactions between the immune system and skeletal muscle play a key role in controlling the progress of both the contusion injury and subsequent muscle repair. Therefore, inflammation is the best target for the development of effective treatments in the recovery of injured muscles 5. Skeletal muscle has the capacity for regeneration after injury. This inflammatory response is followed by muscle repair, regeneration and growth, which involves activation and proliferation of satellite cells, followed by their terminal differentiation 6. Skeletal muscle satellite cells, a population of resident muscle stem cells, are the major contributors to muscle regeneration 7.

Nonsteroidal anti-inflammatory drugs (NSAIDs) are routinely prescribed to alleviate pain-reducing symptoms and restore normal physical function. NSAIDs are exert pain-reducing effects and anti-inflammatory effects by inhibit the activity of cyclooxygenase-1 (COX-1) and COX-2, and accordingly reducing the arachidonic acid (AA) to pro-inflammatory prostaglandins (PGE2 and PGD2) 8. The NSAIDs inhibit both enzyme isoforms, its continuous use can lead to damage to the gastrointestinal tract. So, long-term use of NSAIDs may cause serious side effects such as gastric mucosal damage, which possess multiple side effects and have limited efficacy 9.

Resveratrol (trans-3, 4′, 5-trihydroxystilbene, RES), a natural phytoalexin found in grape skins, peanuts and red wine, has been reported to have a wide range of biological and pharmacological properties 10. Since RES was first isolated in Veratrum grandiflorum and white hellebore plant in the 1940s, scientists have explored RES on a wide variety of health-related issues 11. In recent years, RES has been studied for multiple bio-functional activities in the prevention of cancer 12, asthma 13, cardiovascular disease 14, neurodegenerative diseases 15, diabetes 16, obesity 17, oxidation 18 and inflammation 19. RES exhibits anti-inflammatory effects by downregulating NF-κB, inhibiting the IGF-1R/Akt/Wnt pathway and activating p53, all of which play vital roles in the inflammatory cascade 20.

Supplementation of polyphenols like resveratrol has previously been shown to minimize the deleterious effects of exercise-induced muscle damage and aid in recovery 21. It is therefore likely that RES could also have therapeutic application in the context of inflammatory muscle injury and may be an ergogenic aid for clinically treating muscle injuries after a contusion injury. There has been no prior study to illustrate the physiological effects on RES treatment for contusion-induced muscle injury in mice until now. The objective of this study is to evaluate the potential therapeutic effects of RES treatment on contusion-induced muscle injury in mice.

## Methods

### Animals and Experiment Design

Forty eight-week-old male ICR mice raised under specific pathogen-free conditions were obtained from BioLASCO® (Yi-Lan, Taiwan). All animals were given standard laboratory chow diet (No. 5001; PMI Nutrition International, Brentwood, MO), distilled water *ad libitum* and appropriately housed at the National Taiwan Sport University's animal facility with a 12:12-h light-dark cycle, 22±1ºC and 50-60% humidity. The Institutional Animal Care and Use Committee (IACUC) of National Taiwan Sport University approved all animal experimental protocols and the study conformed to the guidelines of protocol IACUC-10506-M approved by the IACUC ethics committee. All procedures adhered to the American College of Sports Medicine animal care standards.

**Figure 1:**
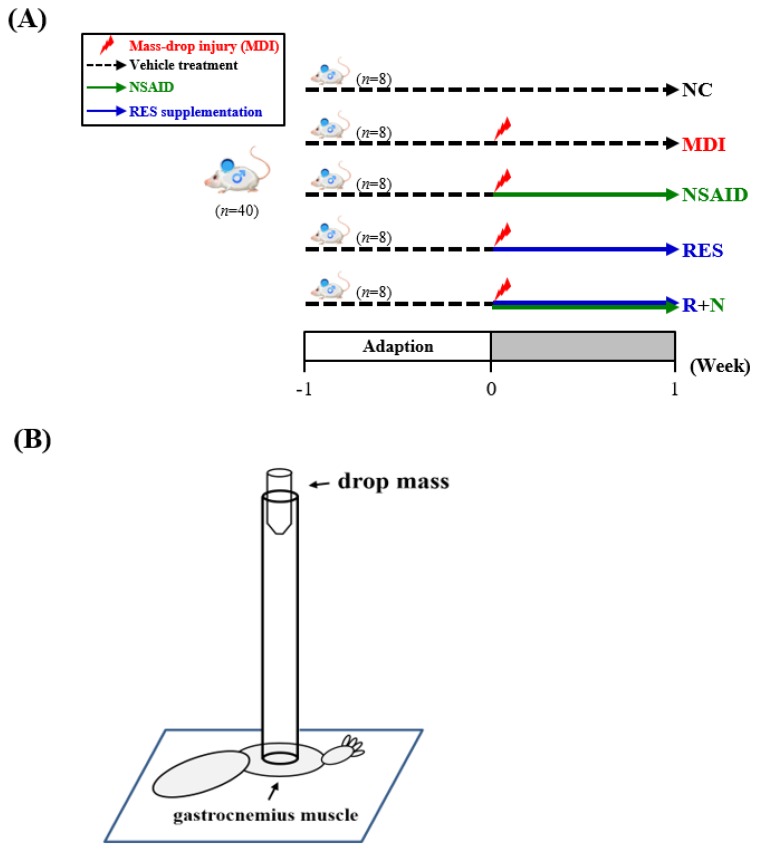
** (A)** The schedule of resveratrol prolotherapy treatment for contusion-induced muscle injuries in mice. **(B)** The gastrocnemius muscle of mice was subjected to a mass-drop injury (MDI).

As shown in Figure 1A, after one week of acclimation, the animals were randomly divided into five groups (n=8 per group in each test): (1) normal control (NC), animals treated with reverse osmosis water (RO) without injury; (2) mass-drop injury (MDI), animals treated with RO water after MDI; (3) MDI + NSAID (NSAID), animals treated with NSAID (diclofenac) after MDI; (4) MDI + resveratrol (RES), animals treated with resveratrol after MDI; (5) MDI + resveratrol+ NSAID (R+N), animals treated with NSAID and resveratrol after MDI. All mice were sacrificed on day 7 after injury, and the liver, kidneys, heart, lungs and gastrocnemius muscles were collected and weighed.

### RES Supplementation and Diclofenac Treatments

The ingredient trans-resveratrol (>98%) used in this study was purchased from Vitacost (Boca Raton, FL, USA). The recommended resveratrol dosage of 25 mg/kg bodyweight for mice, which had been used in our previous exercise fatigue study 22, was administrated by oral gavage for 7 days post-injury.

Diclofenac, a known NSAID and non-specific cyclooxygenase (COX) enzyme inhibitor, was administered at a dose of 10 mg/kg bodyweight for mice by oral gavage for 7 days post-injury. The selected dose is prescribed in clinical practice and does not cause adverse effects 23.

### Induction of Experimental Muscle Contusion Injury and Sample Collection

Mice were anaesthetized with 4%~5% isoflurane. Muscle contusion injury was caused by dropping a 25-g weight from a height of 60 cm (Figure 1B) onto the medial surface of the left gastrocnemius muscle as described in a previous study 24 with slightly modification. This MDI is of medium intensity and does not result in bone injury or gait abnormalities.

### Blood Biochemical Assessments

At the end of the experimental period, all mice were euthanized by 95% CO2 and blood was immediately collected at rest. Serum was collected by centrifugation and the clinical biochemical variables including aspartate transaminase (AST), alanine transaminase (ALT), albumin, blood urea nitrogen (BUN), uric acid (UA), creatinine (CREA), lactic dehydrogenase (LDH) and creatine kinase (CK) were measured using an automatic analyzer (Hitachi 7060, Hitachi, Japan).

### Pathological Histology of Liver and Muscle Tissues

The liver and muscle tissues were removed and fixed in 10% formalin for 24 hours. Tissues were embedded in paraffin and sectioned into 4-μm thickness, stained with hematoxylin and eosin (H&E) and examined with a light microscope equipped with a CCD camera (BX-51, Olympus, Tokyo, Japan) for morphological and pathological characteristics.

### Immunohistochemistry (IHC) on Desmin Expression of Muscle Tissues

The formalin-fixed, paraffin-embedded tissue samples were sectioned into 5-μm thickness. Sections were deparaffinized in two changes of xylene for 10 minutes, rehydrated through an alcohol-to-water gradient, treated with boiling water for 15 min and incubated in 3% hydrogen peroxide for 10 min to block endogenous peroxidase activity. The sections were incubated overnight at 4°C with the rabbit polyclonal antibody desmin (SC-14026; Santa Cruz Biotechnology, Santa Cruz, A, USA) at a working dilution of 1:25). For antigen retrieval, the sections were immunostained with the VECTASTAIN® ABC kit (UNIVERSAL, VECTOR, USA) in accordance with the manufacturer's specifications. The sections were processed with diaminobenzidine (DAB) for staining development and counterstained with hematoxylin.

### Scoring of Immunohistochemical Reactivity

Desmin protein expression was evaluated by immunohistochemistry using Histoscore (H-score), which involves a semi-quantitative assessment of the intensity of staining 25. The proportion-score was then multiplied by the staining intensity from 1 to 3 to provide the final score ranging from 0 to 3, which is the modified H-score (graded as: 0, non-staining; 1, weak; 2, median; or 3, strong) (Figure 5). The range of possible scores was from 0 to 300. Expression level of each component was categorized as low or high according to the median value of H-score.

### Statistical Analysis

Data is expressed as mean ± SEM (n = 8) and significance set at *p* < 0.05. Statistical analysis was done by using one-way analysis of variance (ANOVA) followed by Duncan*'s post-hoc test* for multiple comparisons. All analyses were performed using SAS version 9.4 (SAS Inst., Cary, NC).

## Results

### Effects of Resveratrol Supplementation on Body Weight and Food Intake after Contusion-induced Muscle Injury

The effects of resveratrol supplementation on final body weight and diet intake after contusion-induced muscle injury are shown in Table 1. The initial and final body weights did not significantly differ among the NC, MDI, NSAID, RES and R+N groups. This suggests that contusion-induced gastrocnemius muscle injury of mice does not affect their growth.

The food intake of the MDI, NSAID, RES and N+R groups were significantly lowered by 14.1%, 8.2%, 15.6% and 12.4% (all *p* < 0.0001) respectively, compared with the NC group. The water intake of the MDI, NSAID and RES groups were higher by 18.1% (*p* = 0.019), 29.9% (*p* < 0.0001) and 17.8% (*p* = 0.0221) respectively, compared with the NC group. There was no significant difference between the NC and R+N groups.

### Effect of Resveratrol Supplementation on Organ Weight after Contusion-induced Muscle Injury

Organ weights of experimental animals could provide information about the health status of the mice. As shown in Table 2, there was no difference in the weights of the liver, kidney, heart and lung among the five groups. The relative liver, kidney, heart and lung weight (%) also did not show significant differences between groups. The muscle weight of the NC group was significantly higher (10.6%, *p* = 0.0482) than the RES group. The muscle mass of the damaged leg in the MDI group decreased by 14.8% compared to the NC group (*p* =0.0381). The relative muscle weight (%) of the damaged leg was significant higher in the NC group than the MDI and RES groups by 16.0% (*p* =0.0251) and 15.8% (*p* =0.0251) respectively.

### Effect of Resveratrol Supplementation on Organ Damage Biomarkers after Contusion-induced Muscle Injury

**Figure 2:**
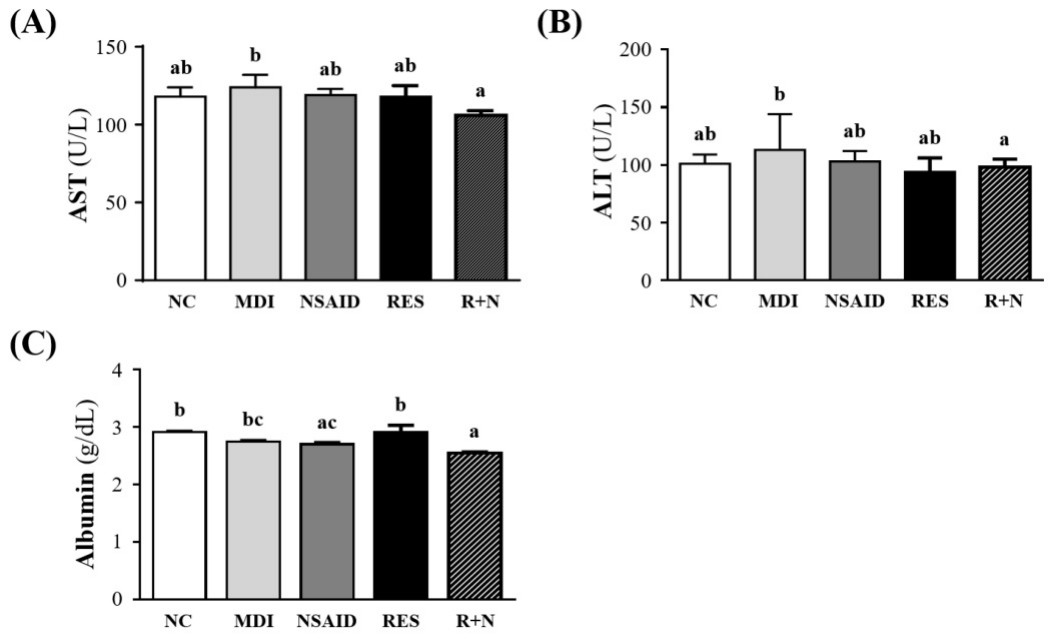
Effect of resveratrol supplementation on organ damage biomarkers after contusion-induced muscle injury. Serum levels of **(A)** AST (aspartate transaminase), **(B)** ALT (alanine transaminase) and **(C)** albumin are shown.

Biochemical results at the end of the experiment could provide clinical information about organ damage biomarkers after muscle damage (Figure 2). The AST and ALT levels were significantly lowered by 14.6% (*p* = 0.0404) and 12.9% (*p* = 0.0414) respectively, in the R+N compared to MDI group. No significant differences were detected between the NC, NSAID and RES groups. Serum albumin level of NSAID and R+N groups were significantly lowered by 7.3% (*p* = 0.0207) and 12.4 % (*p* = 0.0002) respectively, compared with the NC group.

### Effect of Resveratrol Supplementation on Muscle Damage Biomarkers after Contusion-induced Muscle Injury

**Figure 3:**
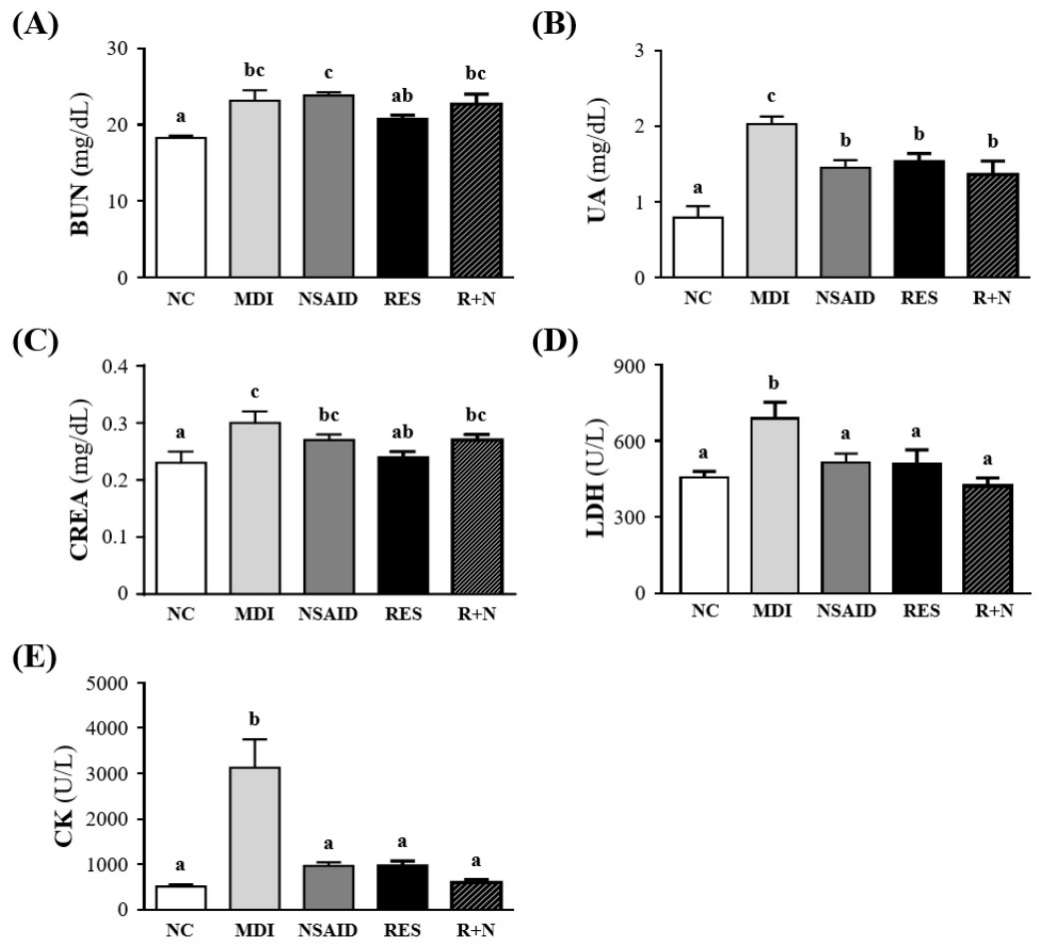
Effect of resveratrol supplementation on the muscular damage biomarkers after contusion-induced muscle injury. Serum levels of **(A)** BUN (blood urea nitrogen), **(B)** UA (uric acid),** (C)** CREA (creatinine),** (D)** LDH (lactic dehydrogenase) and** (E)** CK (creatine kinase).

Figure 3 shows the changes in muscle damage parameters including BUN, UA, CREA, LDH and CK levels. The serum level of BUN in the MDI group showed a 126.6 % increase compared to the NC group (*p* = 0.0005). There were no significant differences between the MDI, NSAID, RES and R+N groups. The serum level of UA in the MDI group showed an increase of 257.1% (p < 0.0001) compared to the NC group. The NSAID, RES and R+N groups had UA levels that were lower by 28.4% (*p* = 0.0042), 24.1% (*p* = 0.0137) and 32.7% (*p* = 0.0012) respectively, than the MDI group. The level of CREA in the MDI group showed an increase of 132.2% (*p* = 0.0009) compared to the NC group. The mice supplemented with RES after injury had significantly lower serum CREA levels by (9.7%, *p* = 0.0058) than the MDI group. Serum LDH level was significantly higher (151.1%, *p* = 0.0008) in the MDI than NC group. After injury, mice treatment with NSAID, RES and R+N had serum LDH levels significantly lowered by 25.2% (*p* = 0.0095), 26.0% (*p* = 0.0077) and 38.7% (*p* = 0.0002) respectively, compared with the MDI group. No significant difference between NC, NSAID, RES and R+N groups was detected. Serum CK activity was significantly higher by 507.4% (*p* < 0.0001) in the MDI than NC group. After injury, mice treatment with NSAID, RES and R+N had significantly lower serum CK activity levels by 69.0%, 68.7% and 80.7% (all *p* < 0.0001) respectively, with no significant difference between NC, NSAID, RES and R+N groups.

### Effect of Resveratrol Supplementation on the Pathological Histology of Liver and Muscle Tissues

**Figure 4:**
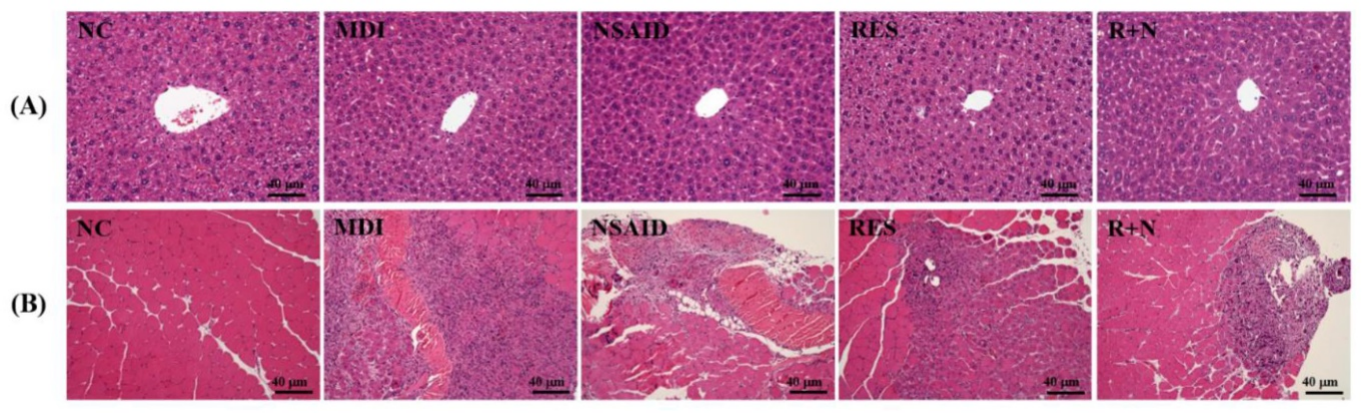
Effects of resveratrol supplementation on pathological histology of the** (A)** liver and **(B)** muscle.

Histological observations of the liver in the MDI, NSAID, RES and R+N groups (Figure 4A) were no different from the NC group. Figure 4B shows the therapeutic effect of RES supplementation against a contusion-induced muscle injury using H&E staining. Histological evaluation of contusion-induced injured muscle tissues revealed that the drop-mass method markedly disrupted and damaged the muscle fibers, causing red blood cells to accumulate in the interstitial spaces. After 7 days of contusion-induced muscle injury, the inflammation of the MDI group was the most serious. Treatment with NSAID and RES restored the disruption of the muscle tissues in the contusion-induced muscle injury. The R+N group exhibited the best recovery condition, suggesting that the therapeutic effect of RES and NSAID together was better than that of RES or NSAID alone.

### Effect of Resveratrol Supplementation on Desmin Expression of Muscle Tissues

**Figure 5:**
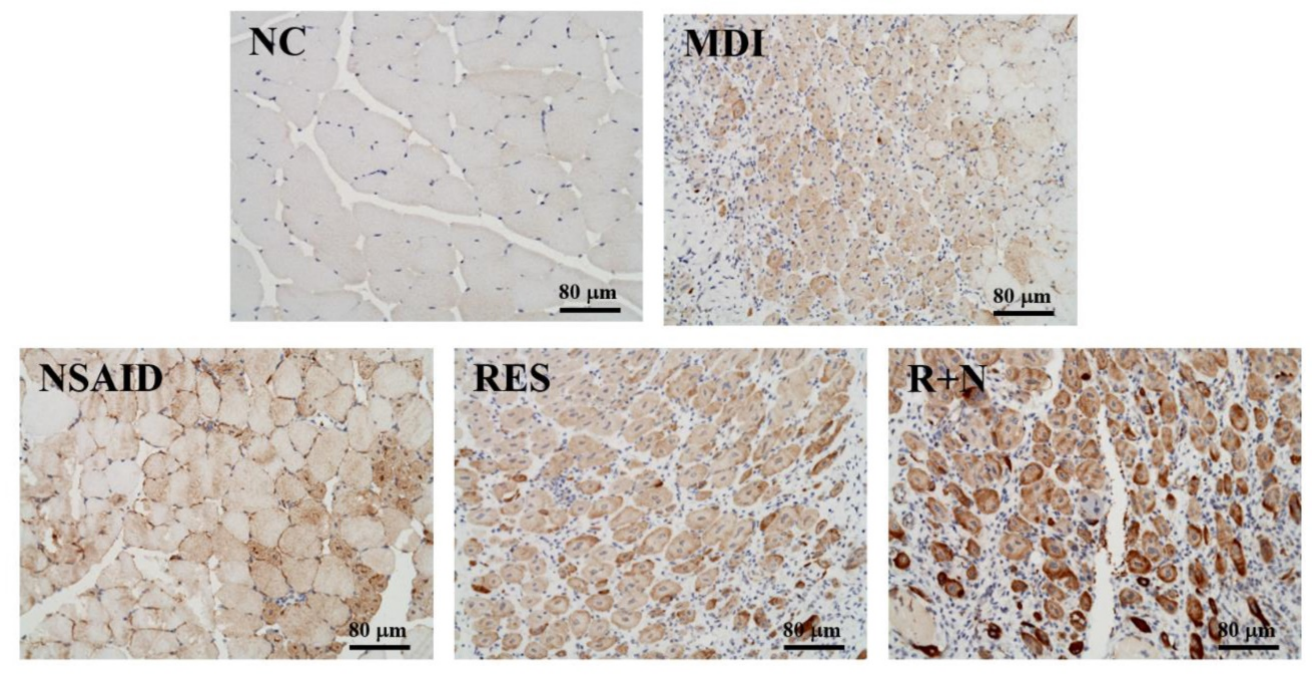
Effects of resveratrol supplementation on IHC staining of desmin protein in muscle tissues.

**Figure 6:**
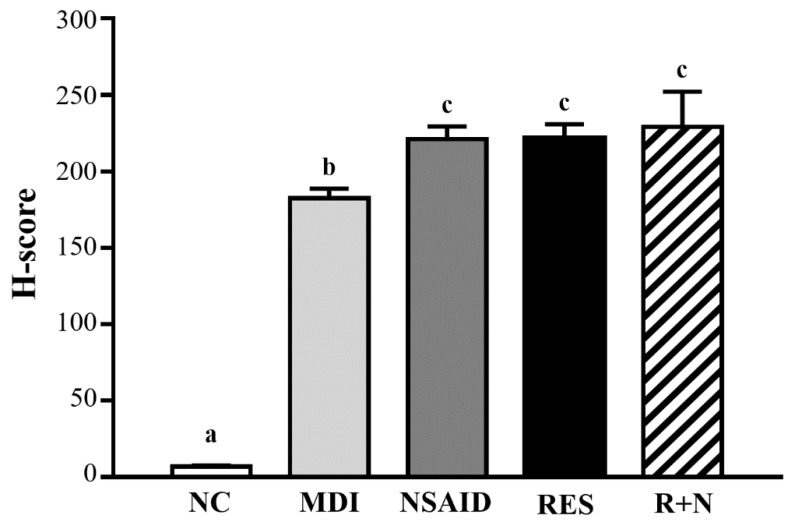
H-score assessments of desmin protein expression in muscle tissues.

As shown in Figure 5, desmin protein expression of the MDI group was increased compared to the NC group. After a contusion-induced muscle injury, mice that were administered RES were able to increase levels of desmin protein, compared with the none-treated group. In addition, expression of desmin protein with RES + NSAID treatment was higher than with NSAID alone. The distribution of H-score is shown in Figure 6. After injury, mice treatment with NSAID, RES and R+N had significantly higher H-score assessments of desmin expression by 121.2% (*p* = 0.0362), 121.5% (*p* = 0.0346) and 125.7% (*p* = 0.0014) respectively, compared with the MDI group. No significant differences were detected between the NSAID, RES and R+N groups. The results indicate that RES supplementation is effective for muscle repair after contusion.

## Discussion

Skeletal muscle injuries are extremely common, amounting to approximately 35% - 55% of all sports injuries 26. Skeletal muscle injuries can result in serious pain, swelling and bruising, ultimately leading to impaired muscle functions 27. When muscles are damaged, chemotactic factors are released by myocytes and other surrounding cells. Monocytes circulating in blood vessels and bone marrow-derived monocytes specially induced and mobilized by the injury; invade the injured tissue and differentiate into pro-inflammatory macrophages 28. Current treatments are directed towards the restoration of full skeletal muscle functions by promoting muscle repair and regeneration, as well as constraining inflammation and muscle fibrosis 29. The treatment options for skeletal muscle injuries so far remain suboptimal, and often result in incomplete recovery.

RES is a natural compound with multiple biological activities. A previous study reported that RES could inhibit various pain and inflammation conditions and able to exert a treatment effect with analgesic and anti-inflammatory activities 30. Exercise-induced muscle damage (EIMD) can cause pain and affect daily activities, such as reduced range of motion, stiffness and swelling 31. In our study, we found that mice significantly decreased food consumption after a contusion-induced muscle injury, but there was no change in body weights between these intervention groups (Table 1). It could be inferred that eating behavioral changes may be caused by pain. The contusion-induced gastrocnemius damage possibly led to pain, affecting food intake status.

Organ weights of experimental animals provide information about the health status, toxicity of treatment and possible physiological effects. The absolute and relative weights of the liver, kidney, heart and lung did not show any significant differences between groups. The wet weights of the gastrocnemius muscle were examined to assess changes in muscle mass during recovery periods. As shown in Table 2, the muscle mass after contusion-induced muscle injury was significantly decreased. A previous study with phytochemical supplementation, such as with curcumin, promoted muscle regeneration and improved behavior associated with delayed onset muscle soreness (DOMS) in mice 32. RES treatment has also been shown to improve skeletal muscle regeneration in aging mice 33. We found that the treatment with RES alone or NSAID alone, did not result in significant muscle mass changes compared with the control group, following a one-week recovery period after muscle damage.

In the present study, levels of organ damage biomarkers of contusion-induced muscle injury such as AST, ALT and albumin were investigated (Figure 2). During strenuous exercise, myocyte injury and necrosis leads to increase in the levels of AST and ALT 34. As shown in Figure 2A and 2B, there was a significant decrease in serum AST and ALT levels in the R+N group, compared with the MDI group. A previous study demonstrated that combining RES with NSAID in treatment could reduce serum AST and ALT activity. This beneficial response was mediated by augmenting the anti-inflammatory effect of NSAID on the injury in rats 35. In this study, the combination treatment of NSAID with RES was able to reduce AST and ALT levels after a contusion-induced muscle injury.

Albumin is one of the most commonly measured serum proteins used to gauge nutritional status 36. Serum albumin can decline in response to physiologic stress and inflammation 37. In our study, the RES group exhibited higher albumin level than the NSAID and R+N groups. This suggests that RES may have a potential benefit in regulating nutritional status to blunt the physiologic stress and inflammatory responses.

Several biochemical parameters have been used to evaluate the extent of muscle injury induced by contusion, such as BUN, UA, CREA, LDH and CK. As shown in Figure 3, the serum levels of BUN, UA, CREA, LDH and CK in the MDI group were significantly increased compared to the NC group. These markers or protein waste products may be naturally elevated following skeletal injuries induced by long term or high intensity exercises 38, 39. At end of a 42-km marathon race, serum levels of myoglobin, CK, and LDH significantly increase according to muscle damage 40. In this study, after the breakdown of skeletal muscle by MDI, the creatinine level in the RES group was significantly lower than the MDI, NSAID and R+N groups. This suggests that myofibril damage is attenuated by RES administration. Higher serum levels of CK indicate that muscle damage is dependent on inflammation, secondary to the mechanical rupture due to excessive repetition of muscle contraction 41.

The activities of CK and LDH have been considered as useful biomarkers for the detection of muscle injury in exercise physiology and sports medicine 42, 43. When a sedentary population suddenly exerts physical activity, the unconditioned individuals usually experience oxidative stress and muscle damage. In sedentary rats, RES administration was able to diminish lipid peroxidation and the elevation of muscle damage markers (CK and LDH) induced by sudden physical exertion. In this study, the serum levels of CK and LDH in the RES, NSAID and R+N groups were significantly lower than those of the MDI group. Resveratrol supplementation has shown protective effects against strenuous exercise-induced oxidative damage and lipid peroxidation, mediated by reduction of LDH, CK, malondialdehyde (MDA), 4-hydroxy-2-nonenal (4-HNE), and 8-hydroxy-2'-deoxyguanosine (8-OHdG) in the serum and muscle 44. Therefore, RES could be used as a treatment for minimizing muscle damage caused by this stress 45. The current results indicate that RES supplementation may decrease muscle damage, similar to NSAID treatment and suggests that resveratrol could further be developed for possible medicinal applications in oxidative stress and muscle damage during intense exercise.

We found no changes in arrangement of sinusoid and hepatic cords with RES and NSAID treatment in mice (Figure 4A). The MDI group (Figure 4B) showed histological changes in the muscle tissue characterized by massive necrosis accompanied by infiltration of inflammatory cells. Only mild or moderate alteration in histology was seen in the RES treatment group. In the MDI, NSAID and R+N groups, increased fibroblast and fibrin depositions were observed in the fibrosis areas. Only a slight fibrosis formation was observed in the RES group.

The levels of the desmin protein indicate the amount of regeneration of muscle satellite cells 46. As shown in Figure 5 and 6, the NSAID, RES and R+N groups had significant increased levels of desmin compared to the MDI group. These results suggest that mice treated with RES could promote desmin expression by facilitating muscle satellite cell regeneration after contusion-induced muscle injury. In contrast, desmin expression in NSAID treatment was significantly lower than RES treatment, implying an inhibitory ability of NSAID toward repair, as suggested by previous literature 28. RES could reduce oxidative stress by inhibiting reactive oxygen species (ROS) production, while reductions in oxidative stress may improve the regenerative outcome and restorative potential of skeletal muscle 47. Resveratrol, a natural antioxidant, mediates anti-apoptotic and anti-catabolic effects on compression injury in skeletal muscle by activation of SIRT1. 48. Previous study showed that SIRT1 could improve muscle mass and functions by enhancing the regenerative capacity of muscles 49. Thus, RES supplementation may have potential effects in increasing skeletal muscle regenerative capacity after contusion-induced muscle injury.

The most common means for dampening the inflammatory response in humans after musculoskeletal injuries is ingestion of NSAID. NSAID exhibit anti-inflammatory effects via inhibition of prostaglandin and reactive oxygen species production as well as suppression of phagocyte migration, aggregation, and other functions 50. Several studies have indicated that NSAID treatment might be beneficial in the recovery from muscle injuryd 51. NSAID work by blocking cyclo-oxygenase (Cox); thus inhibits the synthesis of prostaglandins from arachidonic acid, while one part of the arachidonic acid cascade continues on the lipo-oxygenase pathway 50. On the other hand, NSAID are also known to reduction of satellite cell or myoblast number after injury, which presumably would have a detrimental effect on recovery 52, 53. Resveratrol has been shown to improve cell viability, promote stem cell proliferation and cell repair 54. Therefore, NSAID could be used as a treatment for blocking inflammatory response pathway; RES may could macrophage response and regeneration of muscle satellite cells. In this study, the combination treatment of NSAID with RES was able to potentially accelerate muscle recovery and may be a potential candidate for further development as an effective clinical treatment for muscle repair.

## Conclusions

In conclusion, our results suggest that resveratrol treatment could significantly promote muscle satellite cell regeneration after contusion injury, as evidenced by the increase in levels of desmin protein. In addition, both resveratrol and NSAID treatments alone showed protective effects on contusion-induced muscle damage with decreased levels of serum UA, CREA, LDH and CK. In the muscle damage model by mass-drop injury, administration of resveratrol combined with NSAID showed a better therapeutic effect than NSAID alone in the macrophage response and regeneration of muscle satellite cells. It is possible that the addition of resveratrol treatment limits acute inflammation and increases muscle regeneration after muscle damage. Taken together, our results show that resveratrol may be a potential candidate for more effective muscle repair after contusion injury.

## Figures and Tables

**Table 1:** General characteristics of the experimental groups after contusion injury

Characteristic	NC	MDI	NSAID	RES	R+N
Initial BW (g)	38.58 ± 0.59	38.13 ± 0.56	38.48 ± 0.91	37.04 ± 0.55	37.68 ± 0.68
Final BW (g)	38.88 ± 0.63	39.28 ± 0.69	38.39 ± 0.93	37.79 ± 0.6	37.98 ± 0.67
Food intake (g/day)	7.45 ± 0.10 ^d^	6.40 ± 0.06 ^a,b^	6.84 ± 0.05 ^c^	6.29 ± 0.06 ^a^	6.53 ± 0.08 ^b^
Water intake (mL/day)	9.98 ± 0.14^ a^	12.20 ± 0.85 ^b^	14.25 ± 0.99 ^c^	12.14 ± 0.54 ^b^	9.47 ± 0.06 ^a^

Data are expressed as mean ± SEM for *n* = 8 mice in each group. Values in the same row with different superscript letters (^a, b, c, d^) indicate significant difference (*p* < 0.05) by one-way ANOVA.

**Table 2:** Comparison of different tissues weight after contusion injury and the different treatments

Characteristic	NC	MDI	NSAID	RES	R+N
Liver (g)	2.22 ± 0.08	2.17 ± 0.11	2.14 ± 0.04	2.19 ± 0.07	2.24 ± 0.05
Kidney (g)	0.66 ± 0.02	0.64 ± 0.03	0.64 ± 0.03	0.60 ± 0.02	0.61 ± 0.02
Heart (g)	0.25 ± 0.01	0.26 ± 0.02	0.30 ± 0.09	0.23 ± 0.02	0.21 ± 0.01
Lung (g)	0.22 ± 0.01	0.23 ± 0.01	0.22 ± 0.01	0.21 ± 0.01	0.21 ± 0.01
Muscle (g)	0.38 ± 0.01^a^	0.35 ± 0.01^a,b^	0.36 ± 0.01^ a,b^	0.34 ± 0.02^b^	0.35 ± 0.02^ a,b^
Muscle of normal leg (g)	0.19 ± 0.01	0.20 ± 0.01	0.20 ± 0.01	0.19 ± 0.01	0.19 ± 0.01
Muscle of damaged leg (g)	0.19 ± 0.01^ b^	0.16 ± 0.01^ a^	0.17 ± 0.01^ ab^	0.15 ± 0.01^a^	0.16 ± 0.01^ a^
Relative liver weight (%)	5.70 ± 0.17	5.51 ± 0.23	5.71 ± 0.14	5.67 ± 0.13	5.90 ± 0.09
Relative kidney weight (%)	1.70 ± 0.07	1.63 ± 0.05	1.66 ± 0.07	1.60 ± 0.06	1.62 ± 0.06
Relative heart weight (%)	0.63 ± 0.04	0.67 ± 0.03	0.75 ± 0.20	0.59 ± 0.04	0.55 ± 0.22
Relative lung weight (%)	0.57 ± 0.02	0.58 ± 0.02	0.56 ± 0.02	0.55 ± 0.02	0.56 ± 0.03
Relative muscle weight (%)	0.98 ± 0.03	0.90 ± 0.03	0.94 ± 0.02	0.90 ± 0.04	0.92 ± 0.03
Relative muscle of normal leg weight (%)	0.50 ± 0.02	0.50 ± 0.01	0.51 ± 0.01	0.49 ± 0.02	0.51 ± 0.02
Relative muscle of damage leg weight (%)	0.48 ± 0.02^b^	0.40 ± 0.02^a^	0.43 ± 0.02^a,b^	0.40 ± 0.02^a^	0.41 ± 0.03^a,b^

Data are expressed as mean ± SEM for *n* = 8 mice in each group. Values in the same row with different superscript letters (^a, b^) differ significantly (*p* < 0.05) by one-way ANOVA.
